# The predictive value of uterine artery Doppler in the success rate of pregnancy from the first frozen embryo transfer during the implantation window

**DOI:** 10.1186/s12884-023-06150-y

**Published:** 2023-11-30

**Authors:** Junmei Fan, Junkun Zhang, Suming Xu, Huiping Liu, Weigang Lv, Xingyu Bi, Yanling Liu, Wenjing Shi, Yuxia Zhang, Xueqing Wu

**Affiliations:** 1 Department of Reproductive Medicine Center, Children’s Hospital of Shanxi and Women Health Center of Shanxi, Affiliated of Shanxi Medical University, Taiyuan, Shanxi China; 2 Department of Intensive Care Unit, Third Hospital of Shanxi Medical University, Shanxi Bethune Hospital, Shanxi Academy of Medical Sciences, Tongji Shanxi Hospital, Taiyuan, Shanxi China; 3 Department of Obstetrics and Gynecology, The Third Xiangya Hospital, Central South University, Changsha, Hunan China; 4 Shanxi Medical University, Taiyuan, Shanxi China

**Keywords:** Frozen embryo transfer, Uterine artery Doppler, Implantation window, Ongoing pregnancy rate, Early pregnancy, Pregnancy outcome

## Abstract

**Background:**

Worldwide, frozen embryo transfer (FET) has become a new strategy for the treatment of infertility. The success of FET is closely related to endometrial receptivity. Does uterine artery Doppler during the implantation window predict pregnancy outcome from the first FET?

**Methods:**

A total of 115 retrospectively collected cycles were included in the study, with 64 cycles of clinical pregnancy and 51 cycles of nonclinical pregnancy; There were 99 nonabsent end-diastolic flow (NAEDF) cycles and 16 absent end-diastolic flow (AEDF) cycles. The differences in uterine artery Doppler findings between different pregnancy outcomes were investigated. The clinical pregnancy rate and spontaneous abortion rate in the NAEDF and AEDF groups were compared. The predictive value of uterine artery Doppler during the implantation window in the success rate of pregnancy from the first FET was also investigated.

**Results:**

Between the clinical pregnancy group and the nonclinical pregnancy group, there were no significant differences in the mean resistance index (mRI) (Z = -1.065, *p* = 0.287), mean pulsatility index (mPI) (Z = -0.340, *p* = 0.734), and mean peak systolic/end-diastolic velocity(mS/D) (Z = -0.953, *p* = 0.341); there were significant differences in the mean peak systolic velocity (mPSV) (Z = -1.982, *p* = 0.048) and mean end-diastolic velocity (mEDV) (Z = -2.767, *p* = 0.006). Between the NAEDF and AEDF groups, there was no significant difference in the clinical pregnancy rate (χ2 = 0.003, *p* = 0.959), and there was a significant difference in the spontaneous abortion rate (χ2 = 3.465, *p* = 0.019). Compared with uterine artery Doppler alone, its combination with artificial abortion history, waist-to-hip ratio, LH (Luteinizing hormone) of P (Progesterone) administration day, mPSV and mEDV had a higher predictive value regarding clinical pregnancy from the first FET [ROC-AUC 0.782, 95% CI (0.680–0.883) vs. 0.692, 95% CI (0.587–0.797)].

**Conclusions:**

Uterine artery Doppler, particularly mPSV and mEDV during the implantation window, was useful for predicting clinical pregnancy, and AEDF was related to spontaneous abortion in the first trimester. Uterine artery Doppler combined with artificial abortion history, waist-to-hip ratio, LH of P administration day, mPSV and mEDV have a higher predictive value than uterine artery Doppler alone regarding the pregnancy from the first FET.

## Background

Worldwide, infertility affects at least one tenth of childbearing couples, due to the postponement of marriage age and the change in fertility perception, exhibiting a trend that is increasing [1]. Assisted reproductive technology (ART), represented by in vitro fertilization embryo transfer (IVF-ET), is an effective way to treat infertility. At present, embryo freezing technology continues to improve, and frozen embryo transfer (FET) is gradually increasing in clinical applications, but it varies greatly across regions (27.5–96.3%) [2–4]. FET mainly applies to couples with a selective whole embryo freezing strategy, which carries the risks of fresh embryo transfer, refusal of fresh embryo transfer due to personal factors and preimplantation genetic test (PGT) of couples. Studies have indicated that FET can achieve the same or an even higher clinical pregnancy rate than fresh transfer, while reducing the risks of maternal pregnancy [4–6]. At present, the biochemical pregnancy rate of FET is approximately 15%, the abortion rate is approximately 20%, and the clinical live birth rate is 30–40% [2, 3, 6]. Therefore, it is a hotpot of clinical research to constantly explore effective means of increasing the embryo implantation rate and the pregnancy rate, reducing the biochemical pregnancy rate and spontaneous abortion rate, and closely monitoring the risks of pregnancy.

Uterine artery Doppler was first described by Campbell in 1983, and its importance in obstetrics and reproductive fields has been continuously studied [7]. Uterine artery Doppler results are related to endometrial receptivity and the outcome of embryo transfer [8]. Uterine artery Doppler is an objective way to assess the blood flow state before and after pregnancy. Some studies have suggested that the uterine artery Doppler pulsatility index (PI) has predictive value for endometrial receptivity and pregnancy outcome of fresh embryo transfer [9, 10]. Meanwhile, uterine artery Doppler examination results are closely related to recurrent spontaneous abortion, preeclampsia, foetal intrauterine growth restriction, pregnancy induced hypertension, and other conditions [11, 12]. Some scholars have validated the relationship between uterine artery Doppler results and repeated embryo implantation failure, suggesting that monitoring uterine artery Doppler and giving timely treatment when necessary could improve pregnancy outcomes [13]. However, some scholars believe that uterine artery Doppler does not provide a reliable indication of whether a fresh embryo transfer would result in pregnancy [14]. It is not clear whether uterine artery Doppler examination results during the implantation window can predict pregnancy outcome in patients undergoing the first FET. Therefore, this study retrospectively collected the uterine artery Doppler examination results during the implantion window of patients undergoing their first FET, and analysed their predictive value for pregnancy outcome.

## Materials and methods

### Data collection

The data of patients who underwent FET in the reproductive medicine center of Children’s Hospital of Shanxi and Women Health Center of Shanxi from April 2021 to September 2021, were retrospectively collected. The inclusion criteria were as follows: (1) voluntary uterine artery Doppler monitoring during the implantation window; (2) IVF due to fallopian tube abnormality or mild oligoasthenospermia; and (3) first FET. The exclusion criteria were as follows: (1) maternal age ≥ 38; (2) preimplantation genetic test (PGT) couples; (3) endometriosis or adenomyosis; (4) polycystic ovary syndrome, ovarian reserve decrease and ovulation disorders; (5) uterine malformation or history of tuberculosis, such as intrauterine adhesion, single horn uterus; (6) severe oligospermia, azoospermia and necrozoospermia; (7) ≥ 2 pregnancy loss; (8) hypertension, diabetes, thyroid dysfunction, arrhythmia, tumour disease and other serious diseases.

This study was a retrospective analysis, and was approved by the Medical Ethics Committee of Children's Hospital of Shanxi and Women Health Center of Shanxi (IRB-KYYN-2021–001).

### Study grouping and methods

The cohort of patients was divided into a clinical pregnancy group and a nonclinical pregnancy group, an ongoing pregnancy group and a spontaneous abortion group, and the differences in uterine artery Doppler examination between the two subgroups were analysed. Patients were also assigned to an absent end-diastolic flow (AEDF) group and a nonabsent end-diastolic flow (NAEDF) group, and the differences in clinical pregnancy rate and spontaneous rate were analysed. Ongoing pregnancy refers to pregnancy ≥ 12 weeks, and foetus are viable. The abortion group refers to patients with pregnancy loss that occurs in the first trimester who have a gestational sac that can be observed in the uterine cavity before pregnancy loss. Patients in the biochemical pregnancy group are defined by a serum β- HCG up to 25 mIU/ml (chemiluminescence method), and the absence of an observable gestational sac in or outside the uterine cavity.

After giving progesterone (P) or ovulation, uterine artery Doppler monitoring was performed 1–3 days before the FET. Uterine artery Doppler parameters include: resistance index (RI), PI, peak systolic/end-diastolic velocity (S/D), peak systolic velocity (PSV) and end-diastolic velocity (EDV). Progesterone types in the study included progesterone 40 mg/d (Xianju, Zhejiang, China), intramuscular injection; didroxyprogesterone 40 mg/d (Abbott, Netherlands), oral, combined with progesterone suppository 90 mg/d (Fleet, UK) or 100 mg/d (Dongxin, Hubei, China), and vaginal medication.

### Uterine artery Doppler monitoring

A GE-E8 color Doppler ultrasound instrument produced by GE (United States) was used, and the frequency of the vaginal ultrasound probe was 4–9 MHz. (1) Monitored Doppler parameters of bilateral uterine arteries. The uterine artery is a branch of the internal iliac artery. We detected multiple tortuous arterial branches on coronal sections of the cervix, and the ascending branches supplied the uterus. We selected the branch vessels that ran away from the probe and had rich blood flow. The color Doppler was adjusted appropriately until the blood flow signal displayed well. The width of the sampling door was set to 2 mm, and the sampling window was consistent with the direction of blood flow, with an angle of < 30° with the direction of blood flow [15]. All patients had bilateral uterine arteries measured by a professional at least twice. Generally, 6 continuous and stable cardiac cycle spectra, were obtained enabling blood flow Doppler parameter measurements of the system spectrum, for determining the RI, PI, S/D, PSV and EDV of the bilateral uterine arteries. Their average values, were recorded as the mean RI (mRI), mean PI (mPI), mean S/D (mS/D), mean PSV (mPSV) and mean EDV (mEDV). (2) From 28 days after embryo transfer to 12 weeks of pregnancy, the size, position and morphology of the uterus and gestational sac, were regularly checked, the presence of yolk sac, foetal bud and heart tube pulsation was observed, and the development of the embryo, ovary and pelvic cavity was evaluated.

### Statistical analysis

SPSS 26.0 (IBM, New York) was selected for data analysis. The continuous data are expressed as the median (interquartile range), and the Mann‒Whitney U test was used for difference comparison between subgroups. The categorical data are expressed as numbers/proportions (%), and the Chi-square test was used for difference analysis between subgroups. The receiver operating characteristic (ROC) curve was used to explore the predictive value of single uterine artery Doppler parameters, multiple uterine artery Doppler parameters and combined clinical factors in clinical pregnancy rate from the first FET. *P* < 0.05 was considered statistically significant.

## Results

### General information

**Fig. 1:**
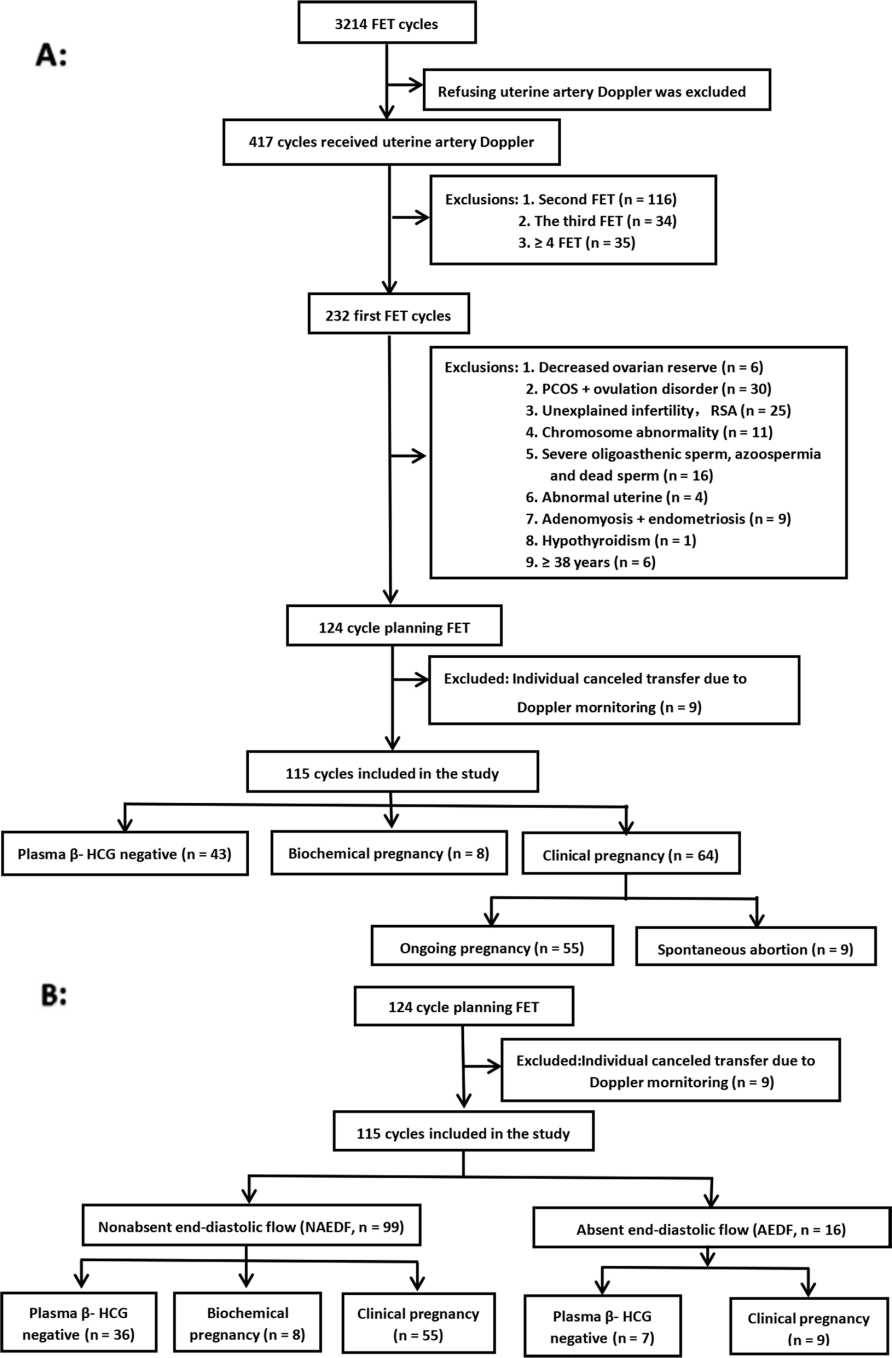
Flow chart. Note:1. There were 8 cycles of biochemical pregnancy included in nonclinical pregnancy, 9 cycle of spontaneous abortion and 1 cycle of heterotopic pregnancy included in clinical pregnancy in part A; 2. In AEDF group, there were 4 cycle of spontaneous abortion and 1 case of ectopic pregnancy

From April 2021 to September 2021, a total of 3214 cycles of FET were carried out in the reproductive medicine center of Children’s Hospital of Shanxi and Women Health Center of Shanxi, of which 417 cycles of uterine artery Doppler monitoring voluntarily proceeded during the implantation window, 1–3 days before embryo transfer. There were 232 cycles that involved a first FET. Excluding those who did not meet the conditions (see the flow chart, Fig. 1), 115 cycles were included in the study.

A total of 115 cycles comprised 64 cycles (55.65%) of clinical pregnancy and 51 cycles of nonclinical pregnancy (including 8 biochemical pregnancies). There were 99 cycles of nonuterine artery Doppler absent end-diastolic flow, the clinical pregnancy rate was 55.56% (55/99), and biochemical pregnancy occured in 8 cycles. There were 16 cycles of uterine artery Doppler absent end-diastolic flow, and the clinical pregnancy rate was 56.25% (9/16). The Doppler absent end-diastolic flow rate of the uterine artery was 13.91% (16/115). Among 64 cycles of clinical pregnancy, 1 cycle (1.56%) was a heterotopic pregnancy, 1 cycle (1.56%) was an ectopic pregnancy, and 9 cycles (14.06%) culminated in spontaneous abortion. Eight biochemical pregnancies were patients without uterine artery absent end-diastolic flow. See Table 1 for details.

**Table 1:** Comparation of general clinical data

General information		NCP	CP	Z/χ^2^	*P*	NAEDF	AEDF	Z /χ^2^	*P*
Age(year)		30(4)	31(4.75)	-1.261	0.207	31(5)	30(4.75)	-1.562	0.118
Infertility time(year)		4(4)	4(4)	-1.913	0.056	4(3)	2(4.75)	-0.915	0.360
Artificial abortion(n)		0(1)	0(0)	-2.065	0.039*	0(0)	0(0)	-1.267	0.205
Spontaneous abortion(n)		0(0)	0(0)	-0.452	0.652	0(0)	0(0)	-0.685	0.493
BMI(kg/m^2^)		22.9(4.30)	24.07(4.99)	-0.909	0.363	23.3(5.01)	24.03(4.62)	-0.554	0.580
Waist-to-hip ratio		0.88(0.05)	0.90(0.04)	-1.974	0.048*	0.89(0.05)	0.88(0.06)	-0.024	0.981
Basal FSH(mIU/ml)		6.95(4.00)	6.9(3.1)	-0.307	0.759	6.81(3.75)	7.2(2.86)	-0.391	0.696
Basal LH(mIU/ml)		2.23(3.22)	2.2(3.65)	-0.286	0.775	2.27(3.18)	2.05(2.78)	-0.292	0.770
Basal E2(ng/ml)		46(33.83)	41(19.8)	-1.125	0.260	40.85(22.15)	46(29.2)	-1.208	0.304
AFC (n)		14(9.00)	15.5(10.00)	-0.757	0.449	14(10)	17(12.5)	-1.625	0.104
Intimal thickness of P day (mm)		9.3(2.00)	9.6(2.6)	-0.887	0.375	9.55(2.28))	9.15(1.88)	-0.053	0.958
LH of P day(mIU/ml)		5.8(8.25)	10.1(9.35)	-2.087	0.038*	8.5(9.8)	11.5(8.8)	-0.760	0.447
E2 of P day(ng/ml)		356.3(271.3)	432.3(232.75)	-0.850	0.395	408.6(255.7)	372.3(214.2)	-0.400	0.689
P of P day(ng/ml)		0.48(0.21)	0.52(0.25)	-0.227	0.820	0.47(0.25)	0.56(0.17)	-2.069	0.039*
Intimal thickness (mm)		9(1)	9(2)	-1.074	0.283	9(1.7)	9.15(2.83)	-0.099	0.921
Transfer embryo count(n)		2(1)	2(0)	-1.320	0.187	2(0)	2(0)	-0.311	0.756
Infertility factor	Fallopian tube	45(88.2%)	44(74.6%)	3.304	0.069	79(84.0%)	10(62.5%)	2.832	0.092^a^
	Abnormal semen	6(11.8%)	15(25.4%)			15(16.0%)	6(37.5%)		
Infertility type	primary	22(43.1%%)	38(59.4%)	2.999	0.083	49(49.5%)	11(68.8%)	2.047	0.153
	secondary	29(56.9%)	26(40.6%)			50(50.5%)	5(31.2%)		
menstrual cycle	regular	42(82.4%)	54(84.4%)	0.084	0.772	80(80.8%)	16(100.0%)	2.419	0.120^a^
	irregular	9(17.6%)	10(15.6%)			19(19.2%)	0(0.0%)		
intima schedule	NC	25(53.2%)	37(60.7%)	0.605	0.437	52(56.5%)	10(62.5%)	0.199	0.655
	HRT	22(46.8%)	24(39.3%)			40(43.5%)	6(37.2%)		
Embyo type	D3 embryo	8(15.7%)	4(6.3%)	2.704	0.100	9(9.1%)	3(18.8%)	0.536	0.464^a^
	blastcyst	43(84.3%)	60(93.8%)			90(90.9%)	13(81.2%)		

1. *NCP* non clinical pregnancy, *CP* clinical pregnancy, *AEDF* absent end-diastolic flow, *NAEDF* nonabsent end-diastolic flow

2. ^a^representing continuous correction of chi square, *representing significant difference

3. P day = on the day of progesterone administration

4. Infertility factors also include 5 couples with two factors

5. In intima schedule, 7 couples adopted the ovulation induction program

### The differences in uterine artery Doppler in different pregnancy outcomes

There were no significant differences in mRI (Z = -1.065, *p* = 0.287), mPI (Z = -0.340, *p* = 0.734) and mS/D (Z = -0.953, *p* = 0.341) between the two subgroups, nonclinical pregnancy (NCP) and clinical pregnancy (CP), for different pregnancy outcomes, and there were significant differences in mPSV (Z = -1.982, *p* = 0.048) and mEDV (Z = -2.767, *p* = 0.006). See Table 2.

**Table 2:** Comparison of the uterine artery blood Doppler between different pregnancy outcomes

Parameters	mRI	mPI	mS/D	mPSV	mEDV
NCP	0.86(0.09)	5.51(5.29)	2.76(1.68)	46.85(15.56)	8.30(3.92)
CP	0.85(0.10)	5.28(4.34)	2.35(2.10)	61.16(17.22)	10.79(5.40)
Z value	-1.065	-0.340	-0.953	-1.982	-2.767
*P* value	0.287	0.734	0.341	0.048	0.006

1. *NCP* nonclinical pregnancy, *CP* clinical pregnancy

2. Biochemical pregnancy was classified as nonclinical pregnancy group

### Comparison of pregnancy outcomes between the AEDF group and NAEDF group

There was no significant difference in the clinical pregnancy rate (χ2 = 0.003, *p* = 0.959), and a significant difference in the spontaneous abortion rate (χ2 = 3.465, *p* = 0.01) between the AEDF group and NAEDF group. See Table 3 and Table 4 for details.

**Table 3:** Comparison of the absent end-diastolic flow rate of uterine artery Doppler between different pregnancy outcomes

Parameters	Nonclinical pregnancy	Clinical pregnancy	χ^2^ value	*P* value
NAEDF	44(86.3%)	55(85.9%)	0.003	0.959
AEDF	7(13.7%)	9(14.1%)		

1. *AEDF* absent end-diastolic flow, *NAEDF* nonabsent end-diastolic flow

2. Biochemical pregnancy was classified as nonclinical pregnancy group

**Table 4:** Comparison of pregnancy outcomes between the NAEDF group and the AEDF group

Parameters	Ongoing pregnancy	Spontaneous abortion	χ^2^ value	*P* value
NAEDF	48(90.6%)	7(63.6%)	3.465	0.019
AEDF	5(9.4%)	4(36.4%)		

1. *AEDF* absent diastolic flow, *NAEDF* nonabsent diastolic flow

2. Biochemical pregnancy was classified as nonclinical pregnancy

3. Continuous correction Chi-square test is selected

### Predictive value of uterine artery Doppler in pregnancy for the first FET

**Fig. 2:**
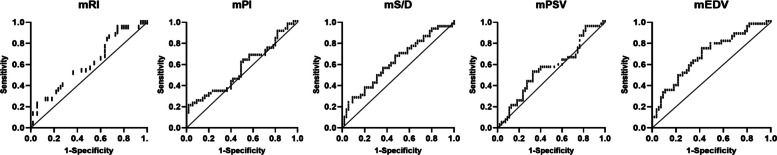
Predictive value of uterine artery Doppler parameters in clinical pregnancy rate from the first FET

Ninety-nine cycles without absent end-diastolic flow of uterine artery Doppler were included to evaluate the predictive value of uterine artery Doppler examination in the pregnancy from the first FET. The predictive value of each index of uterine artery Doppler in clinical pregnancy and the ROC-AUC values were mRI:0.613, mPI:0.559, mS/D:0.555, mPSV:0.619, and mEDV:0.683. The ROC-AUC of mPSV and mEDV were significant (*p* < 0.05). The cut-off values of mRI, mPSV and mEDV were 0.78, 52.06 cm/s and 8.38 cm/s, respectively. See Fig. 2 and Table 5 for details.

**Table 5:** Predictive value of uterine artery Doppler parameters and combing model in pregnancy from the first FET

Paremeters	mRI	mPI	mS/D	mPSV	mEDV	Doppler model	Combing model
AUC	0.613	0.559	0.555	0.619	0.683	0.692	0.782
*P*	0.055	0.317	0.351	0.043	0.002	0.001	0.000
95%CI	0.501–0.724	0.443–0.675	0.440–0.670	0.507–0.732	0.577–0.789	0.587–0.797	0.680–0.883

1. The cut-off value of mRI is 0.77, the cut-off value of mPSV is 52.06 cm/s, and the cut-off value of mEDV is 8.38

2. Doppler model involving five parameters of uterine artery Doppler

3. Combing model involving five parameters of uterine artery Doppler and relevant clinical risk factors in Table 1

### Predicting value of combined parameters in pregnancy from the first FET

**Fig. 3:**
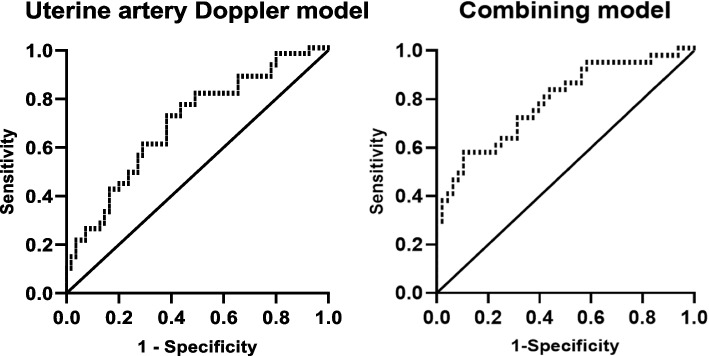
ROC characteristics of uterine artery Doppler and combined multivariate regression in predicting clinical pregnancy from the first FET. Note: 1. Uterine artery Doppler model referring to combining mRI, mPI, mS/D, mPSV and mEDV. 2.Combining model referring to uterine artery Doppler plus relevant clinical risk factors

Five parameters (mRI, mPI, mS/D, mPSV and mEDV) of uterine artery Doppler were introduced into the prediction model, yielding ROC-AUC = 0.692, 95% CI (0.587–0.797). Artificial abortion history, waist-to-hip ratio, Luteinizing hormone (LH) of progesterone administration day, mPSV and mEDV of uterine artery Doppler, were introduced into the prediction model, yielding ROC-AUC = 0.782, 95% CI (0.680–0.883). See Fig. 3 and Table 5 for details.

## Discussion

High-quality embryos and proper endometrial conditions are prerequisites for successful embryo implantation. The aim of endometrial preparation is to ensure the best possible conditions for embryo implantation. With the development of ART, people pay increasing attention to the evaluation of the "best" status of endometrial preparation to ensure the optimum pregnancy outcome. Meanwhile, some scholars propose that the focus of FET should not be simply on the pregnancy rate, but rather on which method is much safer for mothers and foetuses [1].

Uterine artery blood flow plays an important role in endometrial proliferation, luteal phase and pregnancy duration. At present, the clinical uterine artery Doppler parameters include the RI, PI, S/D, PSV and EDV, which enable objective evaluation of uterine artery blood perfusion during the embryo implantation window and different phases of pregnancy, and provide guidance for clinical management. Previous studies indicated that uterine artery Doppler measurements were closely related to recurrent spontaneous abortion, repeated embryo implantation failure, preeclampsia, foetal intrauterine growth restriction, etc., and timely treatment could reduce pregnancy complications and improve maternal and foetal prognosis [10, 12, 16].

Uterine artery blood flow increases from the luteal phase and reaches a peak during the embryo implantation window [17]. The invasion of trophoblasts in the decidua and the formation of placental spiral arterioles in early pregnancy are critical for pregnancy and ongoing pregnancy. This process can be promoted by normal uterine artery blood flow. Uterine vasodilatation and the increase in uterine blood flow in early pregnancy are closely related to pregnancy outcomes [18]. This study shows that there is a significant difference in the mPSV and mEDV of uterine artery Doppler in the implantation window between the clinical pregnancy group and the nonclinical pregnancy group, which hints that blood perfusion of the uterine artery is necessary for embryo implantation. Poor blood perfusion may interfere with endometrial function, change the receptivity of the endometrium, and affect the pregnancy outcome of FET [19]. Some studies also suggested that uterine artery Doppler could be used as an effective index to evaluate endometrial receptivity, and that it was of clinical importance in evaluating pregnancy after retransplantation for patients with recurrent implantation failure [17].

In this study, there was no significant difference in the clinical pregnancy rate between the uterine artery Doppler AEDF group and the NAEDF group. However, there was a significant difference in the spontaneous abortion rate (or ongoing pregnancy rate) between the two subgroups. It might be reasonable to speculate that blood vessels with damaged blood flow can support the energy needed for embryo growth and development in the early stages of embryo implantation. As the embryo develops, it needs more energy. Blood vessels with damaged blood flow are insufficient to support the energy needed by the embryo, and spontaneous abortion occurs. Other studies have shown that the S/D value of uterine artery Doppler in early pregnancy is related to ongoing pregnancy [18]. During pregnancy, uterine artery Doppler findings will also change with the progress of pregnancy, which is of great importance for the prediction and prevention of obstetric complications in early, middle and late pregnancy [10]. In this study, there was no significant difference in the S/D value of uterine artery Doppler between different pregnancy outcomes.

FET is the main part of the clinical application of ART. The preparation of intima is an important guarantee for achieving an ideal outcome. Currently, endometrial preparation mainly includes the natural cycle, ovulation induction cycle and artificial cycle. Most studies have shown that there is no difference in pregnancy outcomes among the three endometrial preparation regimens, but there is a lack of large-scale randomized controlled trials. Some scholars also believe that artificial cycles have the risk of increasing pregnancy complications due to the lack of relevant hormones secreted by the corpus luteum [20]. Therefore, in a certain population, natural cycles, improved natural cycles and ovulation induction cycles are better than artificial cycles [21]. Especially for women with regular ovulation cycles, endometrial preparation should be performed by improving the oestradiol level of dominant follicles in the natural cycle, natural LH peak and natural corpus luteum function, which is the optimum choice [1]. In this study, there was no significant difference in the clinical pregnancy rate between different endometrial preparation schemes, which was consistent with previous research [22]. Some scholars also believe that compared with natural cycles, FET with GnRH agonist followed by oestrogen and progesterone cycles is associated with a higher live birth rate [23].

Increased LH levels may interfere with endometrial receptivity, lowering pregnancy rates even further. Therefore, some scholars suggest that attention should be given to the inhibitory state of the pituitary and follicle during the artificial cycle [24]. This study showed that LH on the day of P administration had an effect on pregnancy outcome (*p* < 0.05). In the natural cycle, if LH is greater than 13 mIu/ml, exogenous HCG is not recommended to stimulate ovulation, otherwise it will have a negative impact on the clinical pregnancy of FET [25]. However, some studies have shown that LH in artificial cycles is not related to pregnancy outcomes [26].

BMI is closely related to pregnancy outcomes [22]. Tunay’s study showed that the waist-hip-ratio is a better predictor of pregnancy outcomes than BMI [27]. This study shows that the difference in the waist-hip-ratio is significant between the clinical pregnancy group and the nonclinical pregnancy group(*p* < 0.05), but the BMI difference between the groups is not significant (*p* > 0.05), which is consistent with previous study [27].

The influence of spontaneous abortion on pregnancy has been recognized by most scholars. Induced abortion will also increase the risk of endometrial damage, and increase obstetric complications [28, 29]. However, it is unclear how induced abortion affects early pregnancy. A previous study showed that a history of abortion was related to pregnancy outcomes [22]. This study shows that the number of induced abortions has an impact on early pregnancy outcomes from the first FET (*p* < 0.05), which might result from endometrial damage.

Uterine artery Doppler has predictive value for preeclampsia and foetal intrauterine growth restriction, but the predictive model combined with clinical factors has a wide range of clinical application prospects [30]. The predictive value of uterine artery Doppler in the pregnancy outcome from the first FET in the study, indicated that combining uterine artery Doppler 5 parameters had higher predictive value than one uterine artery Doppler parameter alone. Meanwhile, uterine artery Doppler findings combined with clinical factors provided the highest predictive value in the success rate of pregnancy from the first FET. To achieve the best pregnancy outcome, clinical factors and imaging examinations should be combined in clinical work.

## Conclusions

In conclusion, the monitoring of uterine artery Doppler during the implantation window is important for achieving ideal pregnancy outcomes and managing patients in early pregnancy from the first FET.

## Data Availability

Availability of data were included in tables and figures.

## Abbreviations

FET: Frozen embryo transfer
NAEDF: Nonabsent end-diastolic flow
AEDF: Absent end-diastolic flow
mRI: Mean resistance index
mPI: Mean pulsatility index
mS/D: Mean peak systolic / end-diastolic velocity
mPSV: Mean peak systolic velocity
mEDV: Mean end-diastolic velocity
ART: Assisted reproductive technology
IVF-ET: In vitro fertilization embryo transfer
PGT: Preimplantation genetic test
ROC: Receiver operating characteristic
