# Correction: The predictive value of uterine artery doppler in the success rate of pregnancy from the first frozen embryo transfer during the implantation window

**DOI:** 10.1186/s12884-026-09221-y

**Published:** 2026-05-23

**Authors:** Junmei Fan, Junkun Zhang, Suming Xu, Huiping Liu, Weigang Lv, Xingyu Bi, Yanling Liu, Wenjing Shi, Yuxia Zhang, Xueqing Wu

**Affiliations:** 1 Department of Reproductive Medicine Center, Children’s Hospital of Shanxi and Women Health Center of Shanxi, Affiliated of Shanxi Medical University, Taiyuan, Shanxi China; 2 Department of Intensive Care Unit, Shanxi Bethune Hospital, Third Hospital of Shanxi Medical University, Shanxi Academy of Medical Sciences, Tongji Shanxi Hospital, Taiyuan, Shanxi China; 3 Department of Obstetrics and Gynecology, The Third Xiangya Hospital, Central South University, Changsha, Hunan China; 4 Shanxi Medical University, Taiyuan, Shanxi China

**Correction: BMC Pregnancy Childbirth 23, 825 (2023)**

**https://doi.org/10.1186/s12884-023-06150-y**

Following publication of the original article [1], the authors identified an error in Fig. 3. The correct figure is given below. The original article has been corrected.

**Fig. 3:**
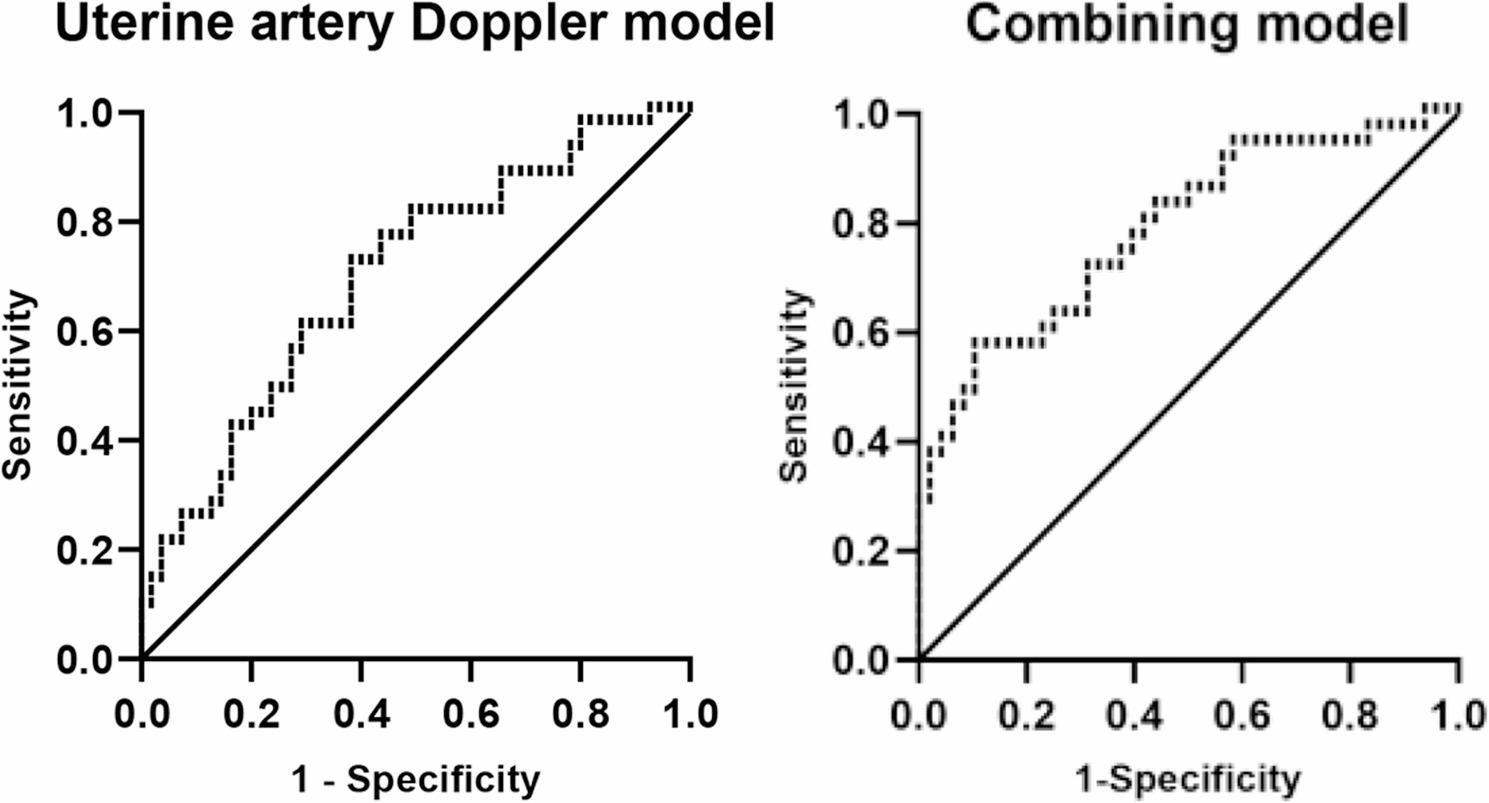
ROC characteristics of uterine artery Doppler and combined multivariate regression in predicting clinical pregnancy from the frst FET. Note: 1. Uterine artery Doppler model referring to combining mRI, mPI, mS/D, mPSV and mEDV. 2.Combining model referring to uterine artery Doppler plus relevant clinical risk factors
